# Tetanus

**Published:** 1895-08

**Authors:** 


### CORRESPONDENCE.

### Tetanus.

Editor Journal of Comparative Medicine:

In your June issue you say "Dr. James A. Waugh has observed that cases of tetanus which are slow in development recover under approved treatment in a much larger per cent., than those which arise rapidly."

Until reading the foregoing I was under the impression that every veterinarian who has seen a sufficient number of cases on which to found a decided opinion was perfectly aware of that fact. By the same token it may also be remarked that cases of tetanus will recover without any treatment if they live long enough. The why and the wherefore are readily explained by the antitoxin theory.

I mean to infer that in cases where the disease is developed slowly the horse gradually becomes accustomed to the tetanic toxins (or toxalbumin, or whatever name may be settled upon as the proper one for the ptomaïnes), and at the same time there is developing in his blood sufficient antitoxin to overcome the effects of the toxins. This power of auto-cure (or recovery as a result of serum-therapy) is better demonstrated in tetanus than in any other infectious disease of which I am aware. Tolerance of a somewhat similar nature is acquired when strychnine, an alkaloid producing an effect similar to that produced by toxalbumin, is given in gradually increased doses; but, of course, strychnine cannot be used to the extent of conferring immunity.

When tetanus is rapidly developed the toxins may have been developed in equally rapid proportion, and in such an amount as to cause death before tolerance can be acquired, or before the antitoxins become sufficient to be of any avail, and consequently all treatment fails; even chloroform will leave the animal in the same condition after he recovers from its anæsthetic effects.

Again, some animals are more susceptible to the influence of certain poisons than others of the same class. We find it almost impossible to put some horses under the influence of chloroform. And it cannot be expected that tetanic toxins can produce their lethal effect on all horses alike. Owing to varying circumstances (too numerous to detail) toxins, like other agents acting on the nervous system, produce symptoms which vary very much in degree in different horses. In some the power of resistance is much stronger than in others; some horses are but slightly affected, while others quickly succumb to the effects of the tetanus bacilli. I have no doubt that some horses enjoy complete immunity.

Now, as to treatment. What is considered approved treatment by one veterinarian may not be approved by another. The expression "approved treatment" is as vague as that other expression so much affected nowadays, "classical treatment." Neither one of the expressions conveys any information whatever. Treatment is judged by its success or failure, and as yet a specific for tetanus has not been perfected. I am of opinion that medicines play but a small part in the cure of this disease; the natural inherent power of resistance possessed by the animal is the great factor in cases that recover. Serum-therapy may eventually place in our hands an agent which will increase the power of resistance when injected into animals that do not possess sufficient power to throw off the effect of the toxins, and until it does rational treatment will mainly consist of a supervision of the general care of the patients, as well as a watchfulness for and a relief of complications.

I practise in a section where tetanus in horses and mules is very frequently met with, in fact the disease often assumes the proportions of an enzoötic, consequently my opportunities for testing the virtue of various lines of treatment are almost unlimited, and up to date I have failed to find a satisfactory medicinal agent for cases that develop rapidly, but I find from exchange of ideas and data with other veterinarians that the number of recoveries under my care is not below the average.

I will append a brief history of a few cases of recent dates. selected from my practice-book, to bring out the points in question.

*March* 19. Black gelding, property of a physician; for about a week had been getting gradually stiffer each day until this date, when owner considered him too stiff to drive. The groom had noticed the membrana nictitans protruding over the eyes for some days past. I found trismus slight, opisthotonos so pronounced that it was with much difficulty he could be backed out of the stall. There was a calk wound on coronet of off forefoot, which had been inflicted about two weeks previously. I cleansed and dressed the wound with a germicide, prescribed potassium bromide in the drinking-water, ordered his usual food and grooming, and walking exercise, one hour at a time, three or four times every day. There was little, if any, change in the symptoms for about ten days, after which time improvement was gradual until April 10th, when he was discharged cured and sent to pasture. To-day he is none the worse for the attack.

*March* 21. Sorrel mare; used to haul wood in a cart. Had developed the disease very gradually for about a week. On this date she was so stiff that she could not pick food from the ground. Trismus slight, opisthotonos pronounced. I found a calk wound on off fore coronet, which I cleansed and dressed with a germicide. Ordered usual food, grooming, and daily walking exercise. Improvement was as gradual as the development. She returned to her usual work on April 4th, and is now in fine condition.

*April* 1. Sorrel gelding; worked on a farm. Did his usual work the day before, and was put away for the night apparently all right. On this date he was taken from the stall with great difficulty, and it was impossible for him to swallow water. I found trismus and opisthotonos intense. There was a contused wound on nigh knee. I told the owner that in my opinion the case was hopeless, but he insisted on the same treatment I had prescribed for a mule of his which recovered from tetanus about a year previous to this case. Therefore I prescribed potassium bromide and cannabis indica, administered *per rectum;* wound was dressed with a germicide. Horse died on April 3d.

*April* 22. Sorrel mare; owned and used by a letter-carrier. I was called to prescribe for her eyes. I found trismus mild, opisthotonos moderate. There were three contused wounds on off hind leg below the hock; which had been inflicted by kicks of another horse about ten days before this date. I dressed the wounds with a germicide, prescribed potassium bromide in drinking-water, and ordered usual care and food, and walking exercise. The symptoms became gradually worse, and on the 26th I prescribed cannabis indica. On the first day of May she was so much worse that she could be moved only at risk of her falling, but I insisted on the exercise being continued. On May 2d she fell on the street while being walked, she struggled much, and it was with great difficulty that she was put on her feet again. Exercise was stopped for two days, but her legs became so œdematous that I insisted on the exercise again, with a man on each side to support her. I discontinued the potassium bromide and cannabis indica, and prescribed nux vomica and ferri sulph. Improvement was very slow. On the morning of May 27th she was discovered cast in her stall. I found her very much exhausted, the effect of her struggles. I prescribed spiritus rectificatus and spiritus ætheris nitrosi, and she quickly recovered from her prostration. On May 30th she was sent to pasture, and is now fully recovered.

*May* 17. Mule; owned by the county and worked in a cart on road repairs. About two weeks previously to this date had received a contused wound on off hock, about a week after which she was noticed to be getting gradually stiffer each day; her principal difficulty was in backing and turning. I found decided trismus, but she could still masticate and swallow her hay and grain; opisthotonos was very pronounced. I dressed the wound with a germicide, prescribed potassium bromide, ordered regular food and daily walking exercise. The legs became œdematous, and the opisthotonos grew daily more intense. On May 31st she got down, and her struggles to regain her feet caused all the symptoms to become aggravated. The workmen from the road were called in, and she was got up and put in a sling. As the swelling of the legs increased, she was taken from the sling daily and walked. The potassium bromide was stopped, and she was given nux vomica and ferri sulphas. Recovery was very gradual, but she is all right now.

*May* 20. Bay gelding; owned and used by a teamster in general hauling. The day before the horse did his usual work, and nothing wrong with him was observed. I found trismus and opisthotonos intense; he could not be moved a step without danger of falling. I made an effort to examine his feet, but the attempt only excited him and intensified the symptoms so that a foot could not be taken from the ground. I pronounced the case hopeless, but the owner insisted on treatment, and I prescribed fluid extract gelsemium, as I thought he might as well waste his money on that drug as any other. There was an ugly saddle-gall on his back which I dressed with a germicide. The horse died the next day.

*June* 6. Bay mare; worked on a farm. Did her usual work the day before and was stabled for the night in her usual condition. I found opisthotonos and trismus very pronounced, but she was still able to eat a mash. She had a large wound on the base of the neck which I dressed with a germicide. Prescribed cannabis indica, *per rectum*, and ordered sloppy mashes. On the 9th she was so much worse that I advised destruction, which advice was ignored. She died on the 10th.

*June* 14. Mule; owned by the city and worked to a cart in the health department. Between two and three weeks previous received a contused wound on off fore fetlock-joint. Had been getting stiffer daily for a week past. The day before in turning he fell, but was helped up and finished his day's work. I found decided opisthotonos and mild trismus. The wound was dressed with a germicide, and I prescribed potassium bromide and cannabis indica, and ordered walking exercise, with his usual food and general care. On the 17th he fell while walking; we raised him and put him in slings. Œdema of the legs and sheath followed, but in other respects he gradually improved. On the 29th the œdema of the sheath and penis was so great that I punctured the swelling in about a dozen places, which was followed by a profuse flow of serum and a decided decrease of the swelling. When each puncture was made he kicked backward viciously with both hind feet, and I concluded that he was able to be taken from the slings for walking exercise. I prescribed nux vomica and ferri sulphas, and discontinued the potassium bromide and cannabis indica. His muscles are still considerably contracted; he walks very stiffly, stumbles, falls, but with help gets up again. I am of opinion he will never die from the effects of tetanic toxins.

*June* 15. Bay mare; property of a transfer company, used for carriage work. Had been thrown off work about ten days previous on account of stiffness and stumbling gait. As she was becoming gradually stiffer the manager sent her to my place. I found mild trismus and moderate opisthotonos. I discovered a punctured wound in off hind foot, which I cleansed and dressed with a germicide, after removing a part of the frog. From the history of the case I concluded that the mare had passed through the worst of the attack, and therefore my instructions simply directed the general care and exercise. On June 30th there was some little stiffness noticeable in the gait, but in other respects the mare was doing well.

*June* 27. Gray mare; used for livery work. About two weeks previous received a punctured wound in foot from a nail. The nail was withdrawn and no other attention paid to the wound. She worked as usual until this date, when she was found to be affected with tetanus. I found trismus and opisthotonos intense. My prognosis was decidedly unfavorable, but I removed the horn from around the puncture and cleansed and dressed the wound with a germicide. I prescribed fluid extract gelsemium and ordered sloppy mashes and walking exercise. On the evening of the 28th her "jaws were set," to use an expression of the colored groom. For professional reasons I declined to visit the patient again. On the 29th a practitioner who did not object to carrying out treatment dictated by the owner and his cronies was called in. I do not know what drugs he prescribed, but I do know the mare died early on the morning of July 1st, just four days after she was attacked. She had the disease to the extent that always kills, no matter what drugs are used.

The foregoing clinical histories, brief as they are, give an idea of the disease as met with by me year in and year out. To be sure, there are times when the disease is more uniformily fatal, or the reverse. I also occasionally meet with variations in its manifestation, for instance, only yesterday I was called to see a case wherein trismus was so intense that the utmost exertion could not separate the upper from the lower incisors an inch, but opisthotonos was scarcely noticeable. The mule walked, backed, and turned very well. My prognosis was unfavorable, for the reason that it was a quickly developed case. I feel certain that in a day or two opisthotonos will be as intense as trismus is now. Respectfully, "OLD DOMINION."

